# miR-3587 Inhibitor Attenuates Ferroptosis Following Renal Ischemia-Reperfusion Through HO-1

**DOI:** 10.3389/fmolb.2021.789927

**Published:** 2022-01-03

**Authors:** Wenqiang Tao, Fen Liu, Jianguo Zhang, Shangmiao Fu, Hui Zhan, Kejian Qian

**Affiliations:** ^1^ Department of Intensive Care Medicine, First Affiliated Hospital of Nanchang University, Nanchang, China; ^2^ Medical Innovation Center, First Affiliated Hospital of Nanchang University, Nanchang, China; ^3^ Department of Infection, First Affiliated Hospital of Nanchang University, Nanchang, China

**Keywords:** ischemia-reperfusion, ferroptosis, acute kidney injury, heme oxygenase-1, microRNA

## Abstract

Renal ischemia-reperfusion (IR) is frequently observed in patients who are critically ill, yet there are no reliable or effective approaches for the treatment of this condition. Ferroptosis, a form of programmed cell death, is regulated by key genes such as glutathione peroxidase 4 (*GPX4*) and heme oxygenase-1 (*HMOX1*) and participates in the injury of renal tubular epithelial cells during IR. This study aimed to investigate the miRNA-mRNA regulatory networks involved in ferroptosis following renal IR. Using bioinformatics analysis, *HMOX1* was found to be significantly upregulated during the early stages of renal IR injury, and microRNA-3587 (miR-3587) was identified as a putative regulator of *HMOX1*. When a miR-3587 inhibitor was applied in a hypoxia-reoxygenation (HR) model system using renal tubular epithelial cells, HO-1 protein (encoded by *HMOX1*) expression was significantly increased relative to that observed in the HR group, with concomitant increases in GPX4 protein levels, enhanced cell viability, a reduction in malondialdehyde content, decreased Fe^2+^ level, and the restoration of normal mitochondrial membrane potential. Transmission electron microscopy showed a reduced or absent mitochondrial crest and a damaged mitochondrial outer membrane. Targeting of *HMOX1* by miR-3587 was confirmed by luciferase reporter gene assay. In conclusion, these preliminary results indicate that inhibition of miR-3587 promotes HO-1 upregulation, thereby protecting renal tissues from IR-induced ferroptosis.

## Introduction

Renal ischemia-reperfusion (IR) is a serious clinical condition that is often encountered in critically ill patients such as those that have undergone major surgery, resuscitation following cardiac arrest, and microcirculation recanalization aftershock, resulting in acute kidney injury (AKI) (Beach et al., 2020). Due to the limited treatment options available for patients suffering from AKI at present, AKI can readily progress to chronic renal insufficiency and even death (Murugan et al., 2021), underscoring the importance of elucidating the pathophysiological mechanisms governing the onset and progression of AKI. AKI is driven by a number of pathophysiological processes, including oxidative stress, microvascular dysfunction, and damage to endothelial cells, all of which result in renal tubular epithelial cell injury (Priante et al., 2019).

Ferroptosis, a recently defined form of regulated cell death, is driven by the iron-dependent peroxidation of membrane lipids (Dixon et al., 2012). Glutathione peroxidase 4 (*GPX4*) and heme oxygenase-1 (*HMOX1*), the key genes of ferroptosis, regulate ferroptosis by controlling the state of lipid peroxidation and the level of iron [(Yang et al., 2014; Tang et al., 2021)]. Recent studies have shown that ferroptosis accounts for most renal tubular epithelial cell death after renal IR (Jiang et al., 2021). The ferroptosis inhibitor XJB-5-131 is able to protect against renal tubular epithelial cell death and consequent kidney injury in a mouse renal IR model (Zhao et al., 2020).

MicroRNAs (miRNAs) are small RNAs that lack coding potential yet can regulate the stability and translation of target mRNAs by binding to complementary sequences within the 3′-untranslated region (UTR) (Lu and Rothenberg, 2018). There is ample evidence that miRNAs can regulate key physiological processes including apoptosis, proliferation, and ferroptosis in a target-specific manner (Zhang et al., 2020). It has been confirmed that hypoxia-reoxygenation (HR) was able to promote the upregulation of miR-182-5p and miR-378a-3p in renal tubular epithelial cells *in vitro*, resulting in GPX4 downregulation and consequent ferroptosis. Consistently, silencing these two miRNAs in an *in vivo* rat renal IR model was sufficient to suppress ferroptosis (Ding et al., 2020). However, further work is required to fully elucidate the regulatory roles of individual miRNAs in the context of ferroptosis to treat AKI. As such, the present study was designed to screen for genes associated with renal IR to identify novel therapeutic targets. Through a series of bioinformatics analyses, *HMOX1* was ultimately identified as a key gene. It has been shown that up-regulation of HO-1, encoded by *HMOX1*, can inhibit ferroptosis in a renal IR model. In the present study, we showed, for the first time, that miR-3587 targets *HMOX1*, and preliminary *in vitro* HR model analyses confirmed that miR-3587 inhibition was sufficient to suppress ferroptosis in renal tubular epithelial cells by promoting HO-1 expression.

## Materials and Methods

### Dataset Selection

**FIGURE 1:**
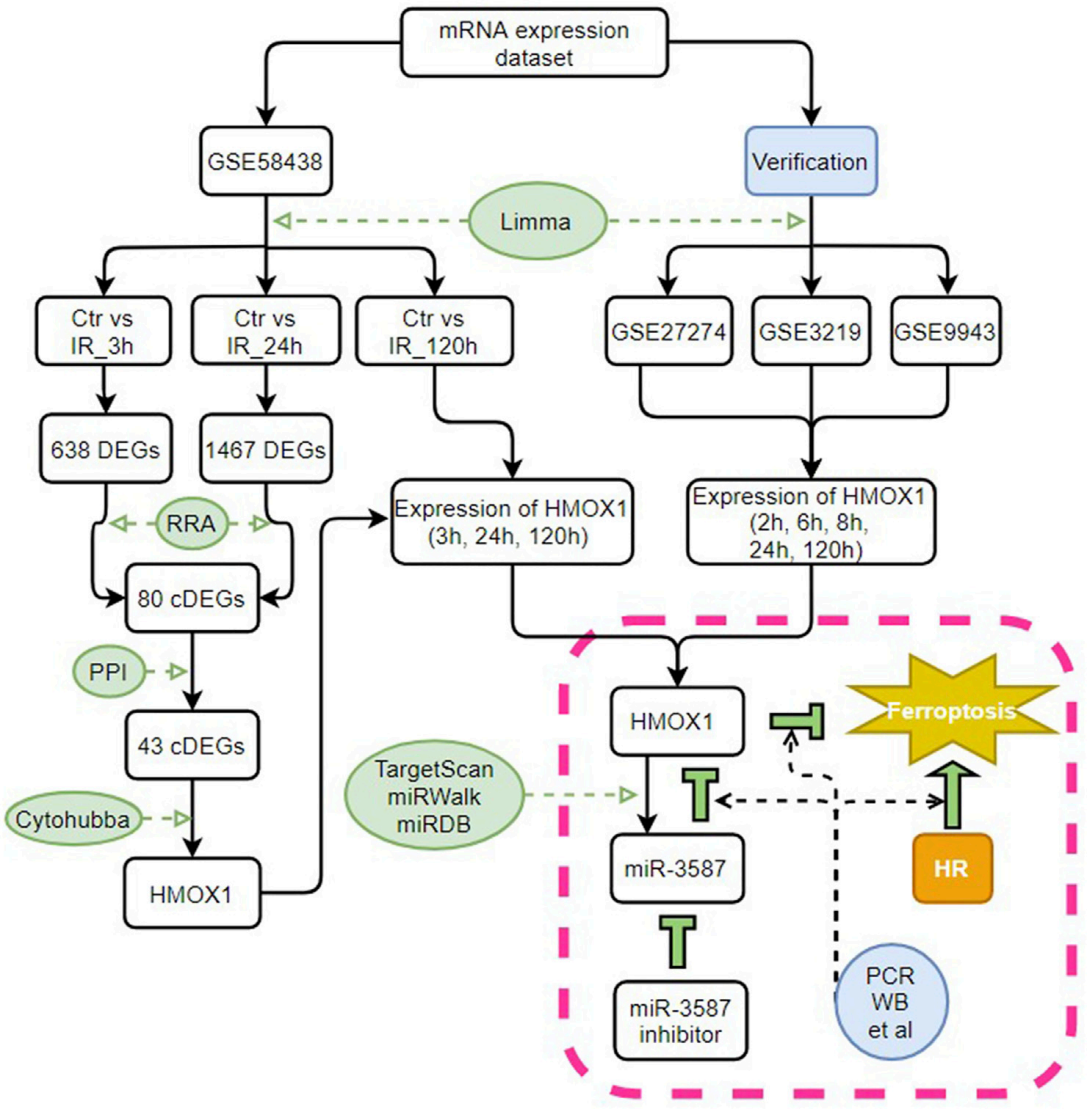
Study flow chart. Ctr, control; IR, ischemia-reperfusion; cDEGs, common differentially expressed genes; RRA, RobustRankAggreg; PPI, protein-protein interaction; HMOX1, heme oxygenase 1; miR, microRNA; HR, hypoxia-reoxygenation; PCR, polymerase chain reaction; WB, western blotting.

The GSE58438, GSE27274, GSE3219, and GSE9943 datasets were downloaded from the Gene Expression Omnibus (GEO) database. These datasets contain information about gene expression in rat tissue; for further information, see Table 1. The data in GSE58438 explored the effects of valproic acid and dexamethasone on acute renal IR injury, while the original purpose of the GSE27274 dataset was to explore the protective effect of upregulated fibrinogen expression on renal IR injury, and samples from the renal cortex and medulla were sequenced separately. The GSE3219 data was from an investigation into early biomarkers of ischemic and nephrotoxic acute renal failure, and the GSE9943 dataset was originally used to explore differential gene expression in the context of the renal IR response in Brown Norway (BN) and Sprague Dawley (SD) rats. In this study, GSE58438 was used for hub gene screening, whereas GSE27274, GSE3219, and GSE9943 were used to confirm the hub gene expression patterns. Samples with additional intervention factors in the GSE58438 and GSE3219 datasets were not included in this study. To control for possible heterogeneity among rat strains, data from BN and SD rats were analyzed separately. For an overview of the study process, see Figure 1.

**TABLE 1:** The details of datasets and the differential expression of HMOX1 at different time points.

Aim	Accession	Platform	Rat	Sample	Exclusion, n	Ctr, n	IR, n	IR time	adj. *p*-value	LogFC
Screening	GSE58438	GPL11534	Wistar	Kidney	27	5	5	I45min/R3h	0.00003635	5.31
							4	I45min/R24h	0.00257	4.24
							5	I45min/R120h	0.1052	1.56
Verification	GSE27274	GPL6101	Wistar	Kidney	0	6	6	I20min/R6h	0.00069931	2.17
							6	I20min/R24h	0.00841,318	1.52
							6	I20min/R120h	0.00947	0.70
Verification	GSE3219	GPL2774	SD	Kidney	17	10	4	I40min/R2h	0.03042455	7.13
							5	I40min/R8h	0.02119530	29.07
Verification	GSE9943	GPL2996	SD	Kidney	0	3	3	I45min/R24h	0.30506	2.29
			BN	Kidney	0	3	3	I45min/R24h	0.99986953	1.44

HMOX1, heme oxygenase-1. SD, Sprague Dawley; BN, Brown Norway. Ctr, negative control. IR, ischemia-reperfusion.

### Common Differentially Expressed Gene Identification

The GSE58438 dataset was initially normalized using the R “limma” package, after which differentially expressed genes (DEGs) between the negative control (Ctr) and IR samples collected at the 3 h (IR_3h) or 24 h (IR_24h) time points were identified, using the following criteria: adjusted *p* < 0.05 and | logFC | ≥1. Then the R “RobustRankAggreg” (RRA) package was utilized to filter the cDEGs between the Ctr vs IR_3h group and the Ctr vs IR_24h group. Volcano plots were constructed using the R “pheatmap” package.

### Functional Enrichment Analyses

The DAVID online tool was used to conduct Gene Ontology (GO) and Kyoto Encyclopedia of Genes and Genomes (KEGG) pathway enrichment analyses and the resultant bubble charts were constructed using the R “ggplot2” package.

### Protein-Protein Interaction Network Construction, Analysis, and hub Gene Verification

The STRING database was used to construct a PPI network incorporating identified the cDEGs using the default parameters, after which the network was imported into Cytoscape software. The top five hub genes were identified using the Cytohubba plugin based on the Degree, Edge Percolated Component (EPC), Density of Maximum Neighborhood Component (DMNC), and Maximal Clique Centrality (MCC) topological measurements. *HMOX1* was identified as the key gene based on the weights of these top five hub genes under each screening method.

The R “limma” package was used to normalize and process the GSE27274, GSE3219, and GSE9943 datasets. *HMOX1* expression was confirmed in all three datasets and for the Ctr vs IR samples collected at the 120 h (IR_120 h) comparison in the GSE58438 dataset.

### Predictive miRNA Identification

The TargetScan, miRWalk, and miRDB databases were utilized to identify putative miRNA regulators of *HMOX1*. Venn diagrams were used to identify miRNAs that were predicted by all three of these databases.

### Dual-Luciferase Reporter Assay

The TransDetect^®^ Double-Luciferase Reporter Assay Kit (TransGen Biotech, Beijing, China; catalog number FR201-01) was used according to the manufacturer’s instructions. The wild-type (Wt) and mutant (Mut) fragments in the 3′-UTR of *HMOX1* related to the miR-3587 binding sites were cloned into the pcDNA3.1 vector. Then, 293T cells transfected with the Relina luciferase plasmid and *HMOX1*-Wt or *HMOX1*-Mut were co-transfected with the miR-3587 inhibitor or microRNA negative control (miR-nc). After 48 h, the luciferase activity was determined in a Varioskan^®^ Flash Full-wavelength scanning multi-function reader (Thermo Fisher, Waltham, MA, United States).

### *In Vitro* Renal IR Model Establishment

Rat renal tubular epithelial NRK-52E cells (Procell, CL-0174) were cultured in high-glucose Dulbecco’s modified Eagle medium (DMEM) containing 1% penicillin and streptomycin (Solarbio, 11965) and 10% fetal bovine serum (FBS, Gibco, 16000-044). Cells in the logarithmic phase of growth were added to culture plates and allowed to attach to the plate surface for 12 h, after which they were transferred to a hypoxic incubator (1% O_2_, 95% N_2_, and 5% CO_2_) for 24 h. The media were replaced and the cells were transferred to a normoxic incubator (21% O_2_ and 5% CO_2_) for 3, 6, or 9 h to establish an *in vitro* HR model of IR injury. Changes in cell morphology were assessed with an Axio Observer 3 microscope (Carl Zeiss, Germany).

### Real-Time Quantitative PCR

RNA was extracted from renal tubular epithelial cells after exposure to hypoxic conditions for 24 h followed by 3, 6, and 9 h of reoxygenation for measurement of *HMOX1* and miR-3587 expression. TRIzol (RNAiso Plus, TaKaRa, Japan, 108-95-2) was used to extract the RNA from the samples, and a NanoDrop 2000 spectrophotometer (Thermo Fisher) was used to assess RNA concentrations and purity. The EasyScript One-Step gDNA Removal and cDNA Synthesis Supermix (TransGen Biotech, AE311-02) were used to prepare cDNA using a StepOnePlus analyzer Real-Time PCR system (Applied Biosystems, United States). All qPCR analyses were performed using a TB Green PreMix Ex Taq TM kit (TaKaRa, RR420A) and an Applied Biosystems 7500 Real-Time PCR instrument. The primer sequences were as follows: β-Actin, positive: 5′-CTA TGA GGG TTA CGC GCT CC-3′ and reverse: 5′-ATG TCA CGC ACG ATT TCC CT-3'; *HMOX1*, positive: 5′-CAG AAG AGG CTA AGA CCG CC-3′ and reverse: 5′-TTG GTG AGG GAAA ATG TGC CA-3′. U6 and miR-3587 primers were synthesized by RiboBio (Guangzhou, China).

### Cell Treatment and Transfection

The miR-3587 inhibitor, miR-nc, and a riboFECT™ CP Transfection Kit were purchased from RiboBio. NRK-52E cells in the logarithmic phase of growth were plated in 6-well plates and subjected to the following four treatment conditions: negative control (Ctr), HR, HR + miR-3587 inhibitor transfection, and HR + miR-nc transfection. Cells were transfected with miR-3587 inhibitor or miR-nc based on the provided kit directions. Briefly, after 12 h when cells were adherent and 50% confluent, the miR-3587 inhibitor or miR-nc transfection mixtures were applied and cells were incubated for 24 h. In this experiment, the HR model system was established as above, with the appropriate transfection mixture being added when the media was changed. For an overview of this experimental protocol, see Figure 4A.

### Western Blotting

RIPA buffer (Applygen, C1053) containing 1% phenylmethanesulfonyl fluoride (PMSF) was used to harvest protein from the treated cells and protein levels were quantified using a BCA kit (Applygen, P1511). Equal amounts of protein (20–30 μg/lane) were then separated on 10% SDS-PAGE and transferred to polyvinylidene fluoride (PVDF) membranes (Millipore, IPVH00010 Immobilon-P Transfer Membrane) with a wet transfer system using 300 mA. Blots were blocked with 5% non-fat milk for 1 h at room temperature, followed by overnight incubation with primary antibodies (HO-1, Abcam, ab68477; GPX4, Abcam, ab125066) at 4°C. According to the antibody instructions, the concentration of the HO-1 and GPX4 antibodies diluted at the ratio of 1:10000 and 1:1,000 were 5 ng/ml and 420 ng/ml, respectively. After three washes with Tris-buffered saline containing Tween 20 (TBST), the membranes were incubated with HRP-conjugated secondary antibodies (ProteinTech, sa00001-2) for 1 h at room temperature. After three additional washes in TBST, Super ECL Prime (US EVERBRIGHT, S6008) was used to detect protein bands together with a Bio-Rad (United States) imaging system. ImageJ v1.8.0 (National Institutes of Health) was used for the densitometric analysis of the protein bands.

### Analyses of Cell Viability, Malondialdehyde Content, Fe^2+^ Level, and Mitochondrial Membrane Potential

Following successful cellular transfection and establishment of the HR model as detailed above, the cell viability, MDA content, and Fe^2+^ level were analyzed using a cell counting kit-8 kit (CCK-8, Abmole, M4839), an MDA determination kit (Nanjing Jiancheng, A003-1–2), and an Iron Colorimetric Assay Kit (Applygen, E1042), respectively, according to the kit instruction manuals.

The NRK-52E cells were placed in a 96-well plate at a density of 4,000 cells per well. Cells were transfected with the miR-3587 inhibitor or miR-nc 24 h before HR induction. Cell viability was measured as described above and absorbances at 450 nm were determined using a Varioskan^®^ LUX Full-wavelength scanning multi-function reader (ThermoFisher).

The MDA concentration was measured by the thiobarbituric acid (TBA) method to evaluate lipid peroxidation. Briefly, treated cells were washed three times with phosphate-buffered saline (PBS), lysed with RIPA buffer containing 1% PMSF for 30 min, and the 0.15 ml lysate was transferred into the sample tube. The blank tube comprised 0.15 ml of distilled water. After addition of 2.5 ml of TBA to each tube and thorough mixing, the tubes were incubated at 100°C for 40 min, cooled under running water, and centrifuged at 4,000 rpm for 10 min. The supernatants were placed in 96-well plates and the absorbances at 532 nm were measured using the multi-function reader. The total protein concentrations were determined with a BCA kit and the MDA level was normalized to that of a sample protein.

The levels of Fe^2+^ were measured as follows: after modeling, the cells were washed three times with PBS, lysed using the supplied lysis solution, and homogenized in a 60 rpm shaker for 2 h. The 3 mM standard supplied with the kit was diluted to concentrations of 300, 150, 75, 37.5, 18.75, 9.38, and 4.69 µM. The buffer was mixed with 4.5% potassium permanganate solution at a 1:1 ratio to prepare mixture A. The standard sample, sample, and diluent of 100 µl were added to the standard, sample, and blank control tubes, respectively, followed by addition of mixture A and thorough mixing. The tubes were incubated at 60°C for 1 h, cooled to room temperature, and immediately centrifuged. Thirty microliters of the Fe^2+^ were added to each tube, mixed well, and incubated for 30 min at room temperature. Two hundred microliter aliquots were then placed in 96-well plates and the absorbance read at 550 nm in the multi-function reader.

Changes in MMP were assessed using an Olympus IX71 fluorescence microscope (Olympus, Japan) using the directions provided with the JC-1 MMP assay kit (Abmole, ab113850). In brief, NRK-52E cells were placed in 24-well plate built-in climbing tablets with a density of 1.5 × 10^5^ cells per well. The cells were transfected with the miR-3587 inhibitor and miR-nc 24 h before HR induction. After modeling, the culture medium was removed and each well was washed once with 500 µl of buffer. One hundred microliters of the supplied kit solution were then added to each well and incubated at 3°C for 10 min. The cells were then washed three times with 500 µl of buffer for each wash for 5 min per wash. A drop of the anti-quenching agent (about 10 µl) was added to the slide, and the round coverslip was inversely covered on the slide, followed by examination and imaging with a fluorescence microscope.

### Transmission Electron Microscopy

Cells were fixed in 2.5% electron microscope-grade glutaraldehyde phosphate buffer for 4 h, rinsed three times with 0.1 M phosphoric acid rinse solution (15 min per wash), fixed in 1% osmic acid solution for 1–1.5 h, and again washed three times with the phosphoric acid rinse solution. The specimens were then dehydrated in step-by-step acetone (50–100%), embedded in Epon812 resin, and cured at 37°C for 24 h, 45°C for 24 h, and 60°C for 24 h. Sections (70 nm) were prepared using a Reichert-Jung ULTRACUT E ultra-thin slicer (Austria), covered copper net, and stained with uranium acetate for 15 min and lead citrate for 15 min. The sections were examined and images using a JEM1200 electron microscope (JEOL, Japan).

### Statistical Analysis

Three biological replicates were used for all experiments. Data are expressed as means ± standard deviations. GraphPad Prism 8.3.0 was used for all analyses. Student’s t-test was used to compare differences between two groups, and ANOVA was used to compare differences between multiple groups. Dunn’s multiple comparisons test was used for one-way ANOVA and Fisher’s least significant difference (LSD) was used for two-way ANOVA. *p* < 0.05 was considered to be significant.

## Results

### Dataset Normalization

Samples included in the GSE58438 datasets were normalized with the R “limma” package (Supplementary Figure S1).

### DEG Identification

**FIGURE 2:**
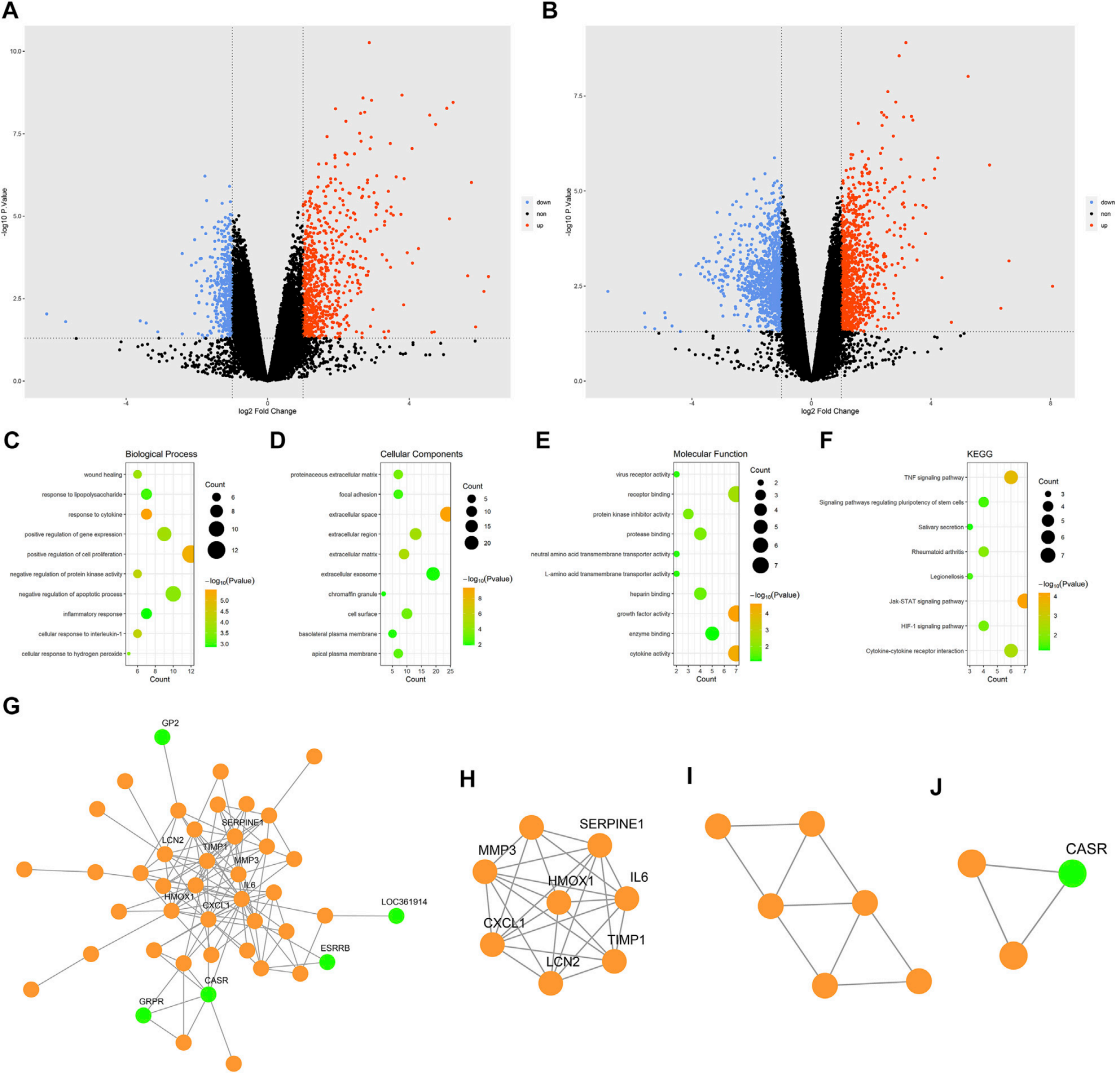
Bioinformatic analysis. **(A)** Volcano plot showing DEGs for the Ctr vs IR_3h comparison. **(B)** Volcano plot showing DEGs for the Ctr vs IR_24h comparison. **(C)** The top 10 GO BP terms. **(D)** The top 10 GO CC terms. **(E)** The top 10 GO MF terms. **(F)** KEGG pathway enrichment results. **(G)** PPI network. **(H)** Functional cluster 1. **(I)** Functional cluster 2. **(J)** Functional cluster 3. Green dots in the G-J panel are used to denote downregulated hub genes.; Abbreviations: DEGs, differentially expressed genes; Ctr, control; IR, ischemia-reperfusion; GO, gene ontology; BP, biological process; CC, cellular component; MF, molecular function; KEGG, Kyoto Encyclopedia of Genes and Genomes.

When analyzing the GSE58438 datasets, 638 DEGs were identified in the Ctr vs IR_3h comparison, including 413 and 225 up- and down-regulated genes, respectively. Additionally, 1,467 DEGs were identified for the Ctr vs IR_24h comparison, including 671 and 796 up- and down-regulated genes, respectively. The DEGs were arranged into volcano plots using the R “ggplot2” package (Figures 2A,B), while clustering heatmaps were drawn using the R “pheatmap” package (Supplementary Figures S2A,B). These heatmaps revealed that one sample in the IR_24h dataset (sample number: GSM1411067) clustered with the Ctr group, suggesting either that the modeling was unsuccessful or that this sample was atypical. To improve the specificity of our overall results, this sample was omitted from subsequent analyses as it had the potential to introduce false-negative experimental results. Following re-screening, a total of 2746 DEGs were identified for the Ctr vs IR_24h, of which 1,304 and 1,442 were up- and down-regulated, respectively.

A total of 80 cDEGs were identified using the R “RRA” package when comparing the Ctr vs IR_3h and Ctr vs IR_24h datasets. Of these cDEGs, 55 and 25 were up- and down-regulated, respectively (Supplementary Figure S2C).

### Functional Enrichment Analyses of cDEGs

GO and KEGG functional enrichment analyses of the 80 cDEGs were performed using the DAVID database. The top 10 biological process (BP), cellular component (CC), and molecular function (MF) GO terms are shown in Figures 2C–E. The top five enriched BP terms were the “response to cytokine”, “positive regulation of cell proliferation”, “cellular response to interleukin-1”, “negative regulation of protein kinase activity”, and “positive regulation of gene expression” categories, while the top five enriched CC terms were “extracellular space”, “extracellular matrix”, “extracellular region”, “proteinaceous extracellular matrix”, and “cell surface”, and the top five enriched MF terms were “growth factor activity”, “cytokine activity”, “receptor binding”, “protein kinase inhibitor activity”, and “protease binding”. These analyses suggested that IR-related genes were primarily associated with responses to the release of inflammatory mediators and the regulation of cell proliferation.

The KEGG pathway enrichment results are shown in Figure 2F, with the top five enriched pathways including the “JAK-STAT”, “TNF”, “cytokine-cytokine receptor interaction”, “rheumatoid arthritis”, and “HIF-1 signaling” pathways. JAK-STAT pathways play critical roles in diverse processes including proliferation and blood production. HIF-1 signal pathway activation regulates cellular responses to hypoxia through the engagement of various compensatory pathways.

### PPI Network Analysis and hub Gene Identification

A PPI network was constructed for the cDEGs using the STRING database. The network included 78 nodes and 124 edges and was visualized using Cytoscape (Figure 2G). The PPI network was analyzed with the MCODE plugin, leading to the identification of three functional clusters (module 1, MCODE score = 7.429; module 2, MCODE score = 3.6; and module 3, MCODE score = 3) (Figures 2H–J). Degree, EPC, DMNC, and MCC values were used to identify the top five hub genes in functional cluster 1 (Table 2). The top five hub genes identified based upon the Degree, MCC, and EPC metrics were identical, and scores for the top five hub genes as screened based upon the Degree and MCC values were identical. Only the DMNC values yielded distinct hub genes. *HMOX1* was a key gene that exhibited the highest EPC score, and it was thus chosen as a target for further study.

**TABLE 2:** Scores for the top five hub gene in functional cluster 1

Sorting	Score					
	Gene	Degree	EPC	MCC	DMNC	Gene
1	HMOX1	7	4.351	840	0.713	TIMP1
2	SERPINE1	7	4.336	840	0.713	LCN2
3	CXCL1	7	4.329	840	0.695	IL6
4	MMP3	7	4.307	840	0.695	HMOX1
5	IL6	7	4.256	840	0.695	SERPINE1

EPC, edge percolated component; MCC, maximal clique centrality; DMNC, density of maximum neighborhood component. HMOX1, heme oxygenase-1. TIMP1, tissue Inhibitor of metalloproteinase-1. SERPINE1, serpin family E member 1. LCN2, lipocalin 2. CXCL1, C-X-C motif chemokine ligand 1. IL-6, interleukin 6; MMP3, matrix metallopeptidase 3.

### Assessment of *HMOX1* Expression in Different Datasets

**FIGURE 3:**
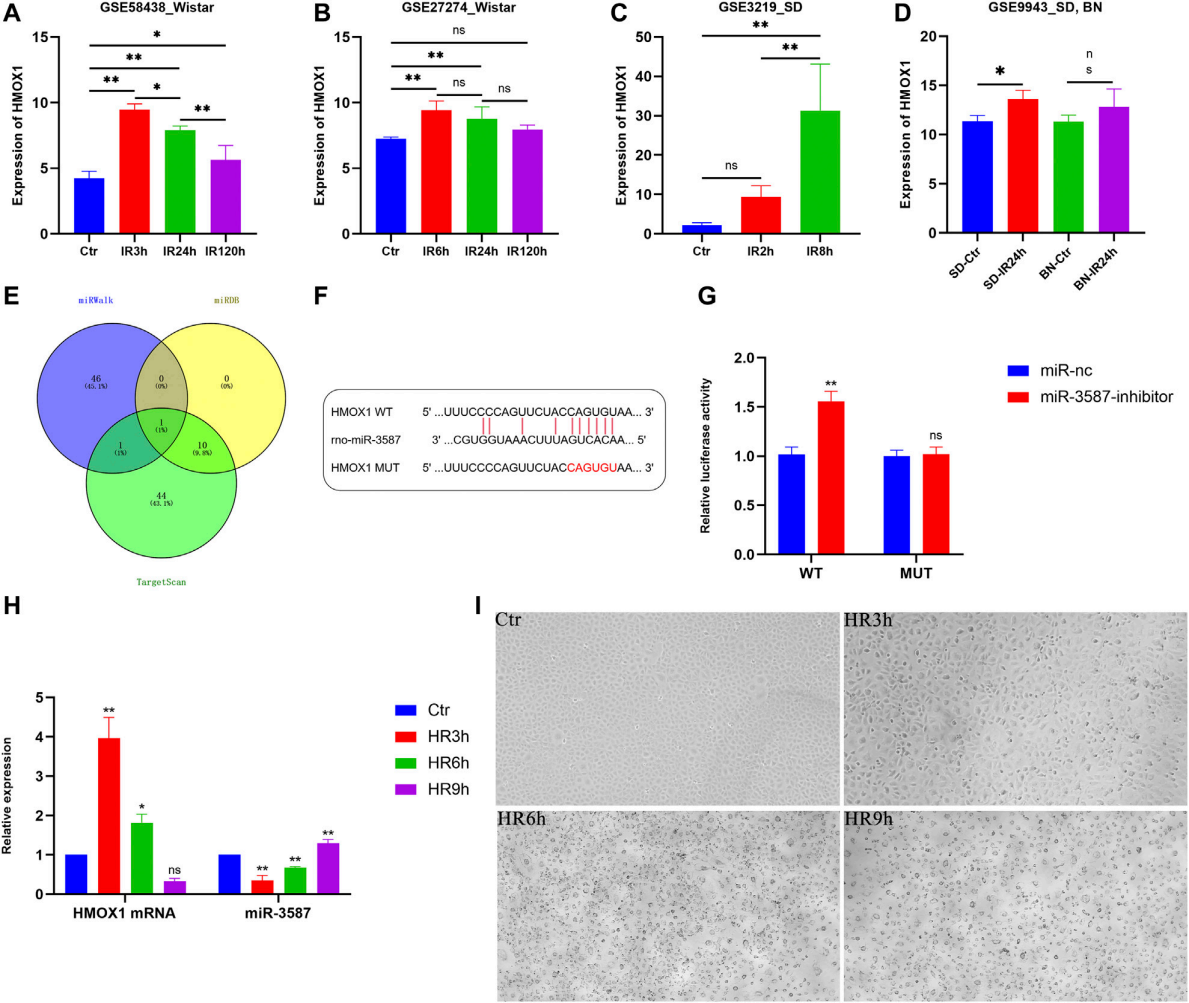
Analysis of *HMOX1* expression patterns in different datasets and *in vitro* verification of the relationship between *HMOX1* and miR-3587. **(A–D)** Trends in *HMOX1* expression in the different datasets. **(E)** Venn diagrams showing miRNAs targeting *HMOX1*. **(F)** Predicted sequence complementarity between miR-3587 and *HMOX1*. **(G)** The luciferase assay showed that the miR-3587 inhibitor, but not the mutant, increased wild-type *HMOX1* mRNA expression. **(H)** PCR-mediated detection of *HMOX1* and miR-3587 expression at different time points following hypoxia-reoxygenation. **(I)** Light microscopy analyses of cell morphology in the Ctr, HR3h, HR6h, and HR9h groups. Scale bar = 4 µm. Magnification ×200. ns, *p* > 0.05; *, *p* < 0.05; **, *p* < 0.01.; Abbreviations: HMOX1, heme oxygenase 1; miR-3587, microRNA-3587; Ctr, control; HR, hypoxia-reoxygenation; SD, Sprague Dawley; BN, Brown Norway.

Samples in the GSE58438 and GSE27274 datasets were from Wistar rats, while samples in the GSE3219 datasets were from SD rats, and samples from the GSE9943 dataset were from both SD and BN rats. *HMOX1* expression in the GSE58438, GSE27274, GSE3219, and GSE9943 datasets is shown in Table 1 and Figures 3A–D. These analyses revealed that *HMOX1* expression rose significantly within 3–8 h after reperfusion, before declining significantly at 24 h post-reperfusion.

### Prediction and Verification of miRNAs Targeting *HMOX1*

Using the TargetScan, miRWalk, and miRDB databases, 56, 48, and 11 miRNAs putatively targeting *HMOX1* were identified, respectively. The only miRNA predicted by all three databases was miR-3587 (Figure 3E). Sequence complementarity between *HMOX1* and miR-3587 is shown in Figure 3F. The direct targeting of *HMOX1* by miR-3687 was confirmed by the dual-luciferase reporter assay. As shown in Figure 3G, compared with the mutant *HMOX1* luciferase reporter, co-transfection of the miR-3587 inhibitor and the wild-type *HMOX1* luciferase reporter resulted in a significant increase in luciferase activity, indicating that miR-3587 binds directly to the 3′UTR of *HMOX1*.

### Trends in *HMOX1* and miR-3587 Expression in Renal Tubular Epithelial Cells Under HR Conditions

To confirm the above results, an *in vitro* renal IR model system was established by incubating NRK-52E under hypoxic conditions followed by reoxygenation for 3, 6, or 9 h qPCR showed that *HMOX1* expression decreased gradually as the reoxygenation time increased, in contrast to miR-3587 expression which was steadily upregulated, with significant differences among groups (Figure 3H). Light microscopic analyses for these cells are shown in Figure 3I. The NRK-52E cells in the Ctr group resembled a cobbled surface. HR3h treatment led to the morphological contraction of the cells, with protrusions from the flattened cobblestone appearance visible. HR6h treatment led to cell membrane rupture and blistering with nuclei of normal size and no evidence of chromatin condensation, the typical morphological changes seen in ferroptosis. HR9h treatment caused the nucleus to deviate to one side and the rest showed vacuolar changes.

### miRNA-3587 Inhibition Enhances HO-1 Expression and Suppresses HR-Induced Ferroptosis in NRK-52E Cells

**FIGURE 4:**
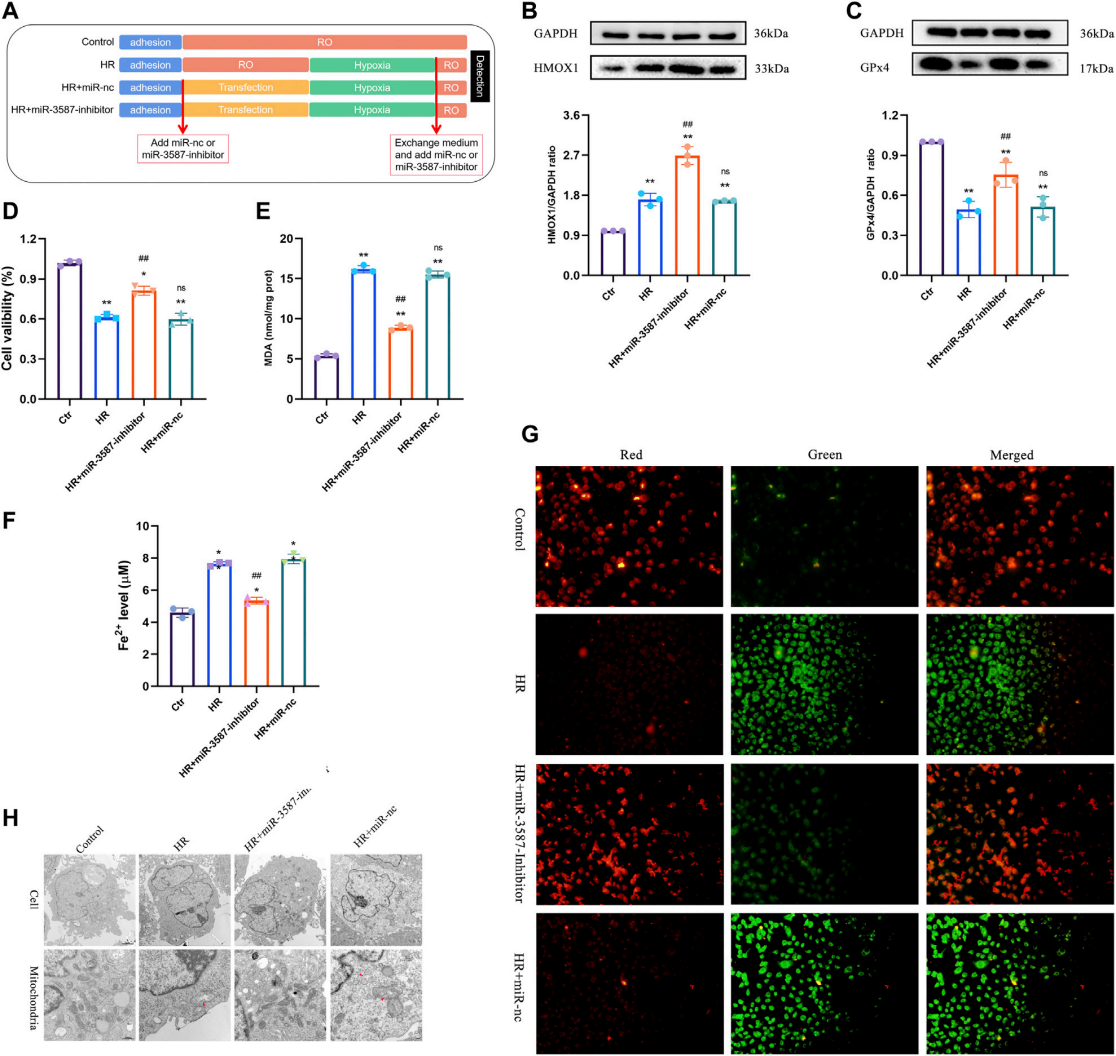
Inhibition of ferroptosis in NRK-52E cells during HR via the miR-3587-mediated regulation of HMOX1. **(A)** Experimental grouping and sample processing. **(B)** HO-1 protein band densitometric analysis. **(C)** GPX4 protein band densitometric analysis. **(D)** Cell viability test results. **(E)** MDA level measurements. **(F)** Fe^2+^ level test. **(G)** Mitochondrial membrane potential was measured using JC-1 in different treatment groups. *p < 0.05; **p < 0.01 vs. control group. ##*p* < 0.01; ns, *p* > 0.05 vs. HR group. **(H)** Transmission electron micrograph showing morphological changes in mitochondria. Abbreviations: HR, hypoxia-reoxygenation; RO, reoxygenation; miR-nc, microRNA negative control; miR-3587, microRNA-3587; Ctr, control; HMOX1, heme oxygenase 1; GPX4, glutathione peroxidase 4; MDA, malondialdehyde.

Details of the experimental conditions and sample collection methods are outlined in Figure 4A. The results of Western blotting indicated significant increases in HO-1 expression in the HR group, while treatment with the miR-3587 inhibitor resulted in further increases in HO-1 expression (Figure 4B). *GPX4* is an important regulator of ferroptosis, and transfection with the miR-3587 inhibitor was associated with increased GPX4 expression (Figure 4C). The effects of miR-3587 on NRK-52E cell viability were assessed using the CCK-8 assay, showing that miR-3587 inhibition was associated with increased viability (Figure 4D). MDA is a lipid peroxidation by-product that reflects the degree of lipid peroxidation in cells, thereby offering indirect insight into ferroptosis induction. The MDA levels in HR-induced NRK-52E cells declined significantly following miR-3587- transfection (Figure 4E), as did the level of Fe^2+^ (Figure 4F). Ferroptosis is associated with significant reductions in MMP. When the MMP is high, JC-1 aggregates in the mitochondrial matrix to form a polymer that fluoresces red. When the MMP is low, JC-1 cannot accumulate in the mitochondrial matrix and is seen as a green-fluorescing monomer. The Ctr group showed red JC-1 fluorescence, whereas green fluorescence was observed following HR treatment, and miR-3587-inhibitor treatment normalized MMP in these HR-treated cells, which exhibited red fluorescent signals (Figure 4G). As shown in Figure 4H, the mitochondrial ridge was reduced or had disappeared, while the mitochondrial outer membrane was damaged in the HR and HR + miR-3587-nc groups, the typical mitochondrial changes associated with ferroptosis.

## Discussion

The kidneys are highly sensitive to insufficient perfusion, with renal IR often occurring in response to shock, trauma, transplantation, and other factors in critically ill individuals, resulting in the occurrence of AKI in 5–20% of these patients (Bagshaw et al., 2010). Acute renal tubular epithelial cell necrosis is a primary pathological finding in AKI (Deng et al., 2019). In the absence of timely intervention, small abnormalities in serum creatinine levels can result in severe complications or mortality (Uchino et al., 2006). Previous studies have shown that ferroptosis is one of the important mechanisms of renal tubular epithelial cell injury in IR (Linkermann et al., 2014). As a unique method of programmed cell death, ferroptosis is essentially a metabolic disorder of lipid oxides in cells, which are over-produced through the catalytic action of iron ions (Dixon et al., 2012). Weakening of the cell’s antioxidant capability and a resultant accumulation of lipid-reactive oxygen species results in an imbalance of the intracellular redox state, leading to the induction of cell death. The biological characteristics of ferroptosis include increased production of reactive oxygen species, aggregation of iron ions, and ultrastructural changes including cell membrane rupture and blebbing, mitochondrial atrophy, decreased or absent mitochondrial cristae, increased membrane density, and normal nuclear morphology without chromatin agglutination (Stockwell et al., 2017). The process of ferroptosis involves a variety of mechanisms and is precisely regulated by multiple signaling pathways (Bersuker et al., 2019). Further study regarding the role of ferroptosis in different diseases is warranted to identify novel therapeutic targets and to guide drug development.

Recent advances in genetic sequencing have highlighted a number of genetic changes associated with the progression of different diseases, enabling researchers to gain new insight into the etiological basis for early-stage renal IR injury and thus to develop novel treatments for AKI. Here, we employed a bioinformatics approach, leading to the identification of *HMOX1* as a key gene with miR-3587 predicted to target *HMOX1*. Inhibition of miR-3587 was sufficient to decrease ferroptosis in renal tubular epithelial cells subjected to HR treatment by promoting HO-1 upregulation. It is worth noting that, on the one hand, the present study only screened the cDEGs of Ctr vs IR_3h and Ctr vs IR_24h to screen the early genetic changes of IR. On the other hand, with reference to other research methods [(Luo and Ma, 2021; Yang et al., 2020)], the present study used external validation datasets which utilized different rat models, all of which exhibited comparable HMOX1 expression trends across diverse sample types. It is reported that HMOX1 plays a critical role in the HIF-1 signaling pathway, which is consistent with the results of bioinformatics analysis in this study, providing a theoretical basis whereby HMOX1 may improve IR injury outcomes (Guo et al., 2019). In addition, this study is the first to report the use of miRNAs targeting HMOX1 to regulate ferroptosis in a renal IR model system.

HO-1 is an inducible enzyme that catalyzes heme conversion into biliverdin, carbon monoxide, and free ferrous ions during oxidative stress. From a mechanistic perspective, HO-1 can also release carbon monoxide and free iron ions, thereby aggravating oxidative stress (Tang et al., 2021). The upregulation of HO-1 is evident in many different human malignancies, where it plays an important role in regulating the stability of the tumor microenvironment in a manner that promotes tumor cell proliferation, angiogenesis, and metastasis (Tang et al., 2021). However, HO-1 can also promote ferroptosis in cancer cells in therapeutic contexts (Lin et al., 2021). There is also evidence that HO-1 primarily plays a beneficial role in IR injury (Hou et al., 2020). Pei et al. demonstrated that normobaric hyperoxia can promote HO-1 upregulation, thereby protecting against renal IR injury (Hou et al., 2020). Su et al. reported that Panx1 gene deletion can protect against IR-induced renal cell ferroptosis in a mouse model system by promoting HO-1 upregulation (Su et al., 2019). In the present analysis, HMOX1 upregulation was observed in four datasets during the early stages of renal IR before decreasing significantly at 24 h, although no significant difference was observed in the Brown Norway rat model in the GSE9943 dataset at the 24 h timepoint. HMOX1 expression levels were also confirmed *in vitro*, with expression being significantly lower at 9 h post-HR relative to 6 h post-HR. Renal function changes continuously following reperfusion (Basile et al., 2005), such that animals ultimately develop secondary chronic kidney disease after reperfusion (Matsushita et al., 2021). As such, approaches to regulating ferroptosis and other early stages of early renal IR injury are critical to improving outcomes.

miRNAs can serve as key regulators of gene expression (Lu and Rothenberg, 2018), and both miRNA mimics and inhibitors have been highlighted as promising therapeutic tools in preclinical contexts (Tang et al., 2017). It has been reported that miR-15a-3p interacts with *HMOX1* to inhibit the metastasis of hepatocellular carcinoma. However, of the three predictive websites used in this study, only TargetScan predicted that it targeted *HMOX1* directly [(Jiang et al., 2020)]. Studies have shown that ischemia-reperfusion conditions result in the upregulation of miR-182-5p and miR-378a-3p, activating ferroptosis by downregulating *GPX4* and *SLC7A11* (Ding et al., 2020). However, there have been relatively few prior analyses of how miRNAs modulate ferroptosis in the context of renal IR. We identified miR-3587 as a predicted regulator of *HMOX1* through three online databases based upon shared sequence complementarity, which was verified by the dual-luciferase reporter assay.

In earlier studies, HO-1 was shown to be significantly upregulated under ischemic conditions, with significant increases in SD rats treated with cobalt protoporphyrin leading to reductions in oxidative stress, inflammation, and renal injury (Barakat et al., 2018). Following the observed HO-1 upregulation in IR, a miR-3587-inhibitor was transfected into cells to promote further HO-1 upregulation to explore its beneficial effects in this pathological context. *GPX4* is a key ferroptosis-related enzyme and the only enzyme capable of detoxifying lipid peroxides. The overexpression or knockout of *GPX4* alters the rates of lethality associated with different inducers of ferroptosis (Yang et al., 2014). Herein, we established an *in vitro* HR model using renal tubular epithelial cells and found that transfection with a miR-3587 inhibitor was sufficient to significantly increase HO-1 expression, enhance cell viability, reduce MDA and Fe2+ levels, and augment *GPX4* expression. In ferroptosis, mitochondria typically shrink, and the cristae become less prominent or disappear entirely (Dixon et al., 2012). Here, JC-1 and TEM were utilized to detect changes in MMP and mitochondrial morphology, indicating that miR-3587 inhibitor treatment attenuated mitochondrial damage.

There are several limitations to the present study. First, the sample size was relatively limited, as even though four datasets were included in the study, the sample size for each individual dataset was small. Further follow-up studies of murine and human samples are thus warranted to confirm and expand upon these results. Second, the *in vitro* verification approaches used were not sufficiently comprehensive owing to technical limitations and time considerations. In principle, a miRNA inhibitor combines with miRNA to inhibit the role of the miRNA rather than changing the expression of the miRNA. Thus, the effects of the miRNA inhibitor can be verified only by detecting the protein expression level of the target gene. Hence, further approaches based on shRNA and antagomir to modulate *HMOX1* and miR-3587 expression should be considered to further understand the regulatory roles of these genes in the context of renal IR injury.

## Conclusion

Herein, a bioinformatics approach was employed leading to the identification of *HMOX1* as a key regulator of renal IR injury through its targeting by miR-3587. By establishing an *in vitro* renal IR model, based upon characteristic ferroptosis-related changes including the protein levels of HO-1 and GPX4, cell viability, the degree of lipid peroxidation, the mitochondrial membrane potential, and the mitochondrial morphology of NRK-52E, we preliminarily explored a mechanism whereby inhibiting miR-3587 can prevent IR-associated ferroptosis in renal tubular epithelial cells at least in part by promoting HO-1 upregulation.

## Data Availability

The datasets presented in this study can be found in online repositories. The names of the repository/repositories and accession number(s) can be found below: https://www.ncbi.nlm.nih.gov/, GSE58438 https://www.ncbi.nlm.nih.gov/, GSE27274 https://www.ncbi.nlm.nih.gov/, GSE3219 https://www.ncbi.nlm.nih.gov/, GSE9943.
