# Supplementary figures and images for: miR-3587 Inhibitor Attenuates Ferroptosis Following Renal Ischemia-Reperfusion Through HO-1
Source: Front Mol Biosci. 2022 Jan 3;8:789927. doi: 10.3389/fmolb.2021.789927 (PMC8762253; doi:10.3389/fmolb.2021.789927)

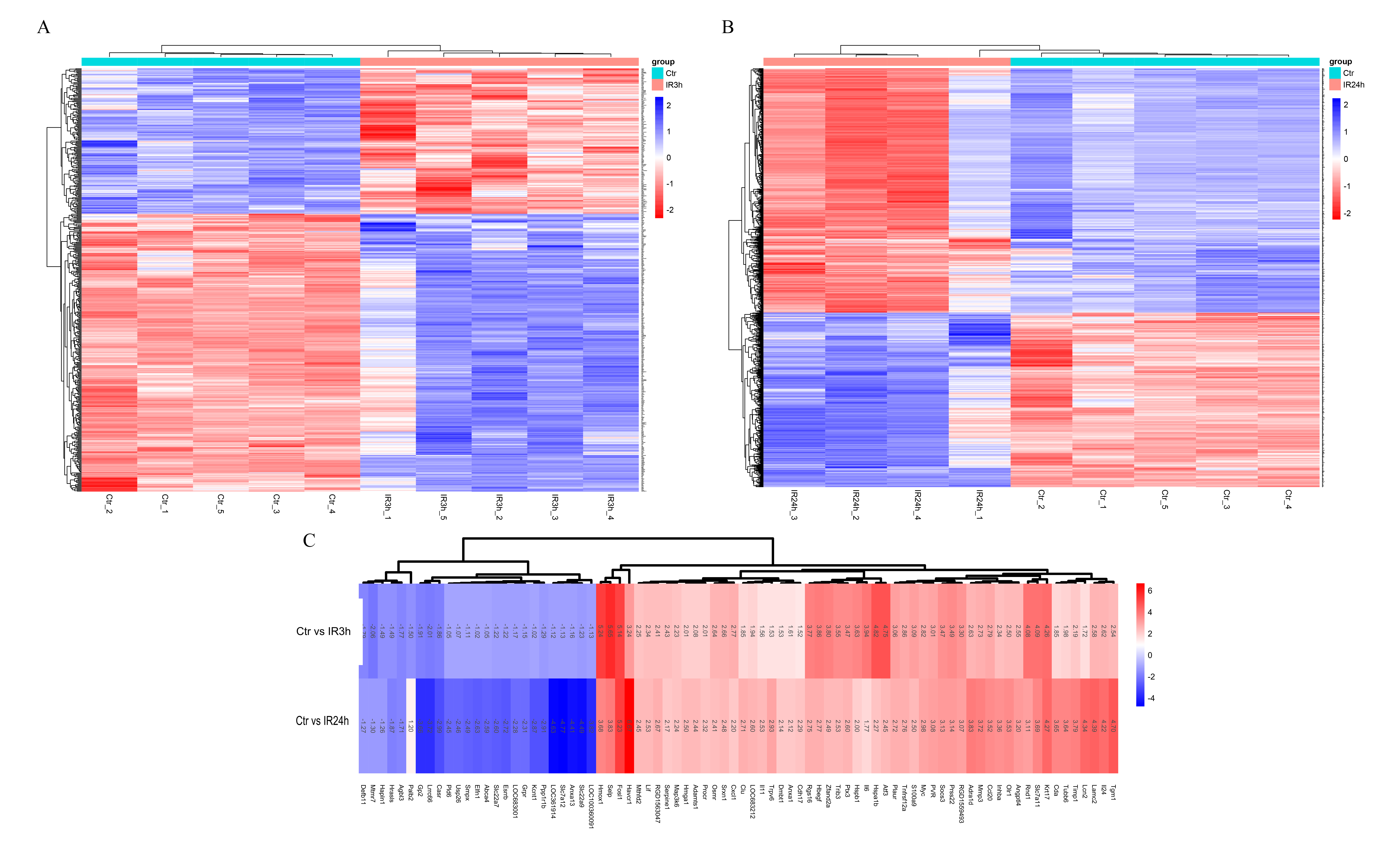

Supplement: Supplementary file 1 [file Image2.TIF]

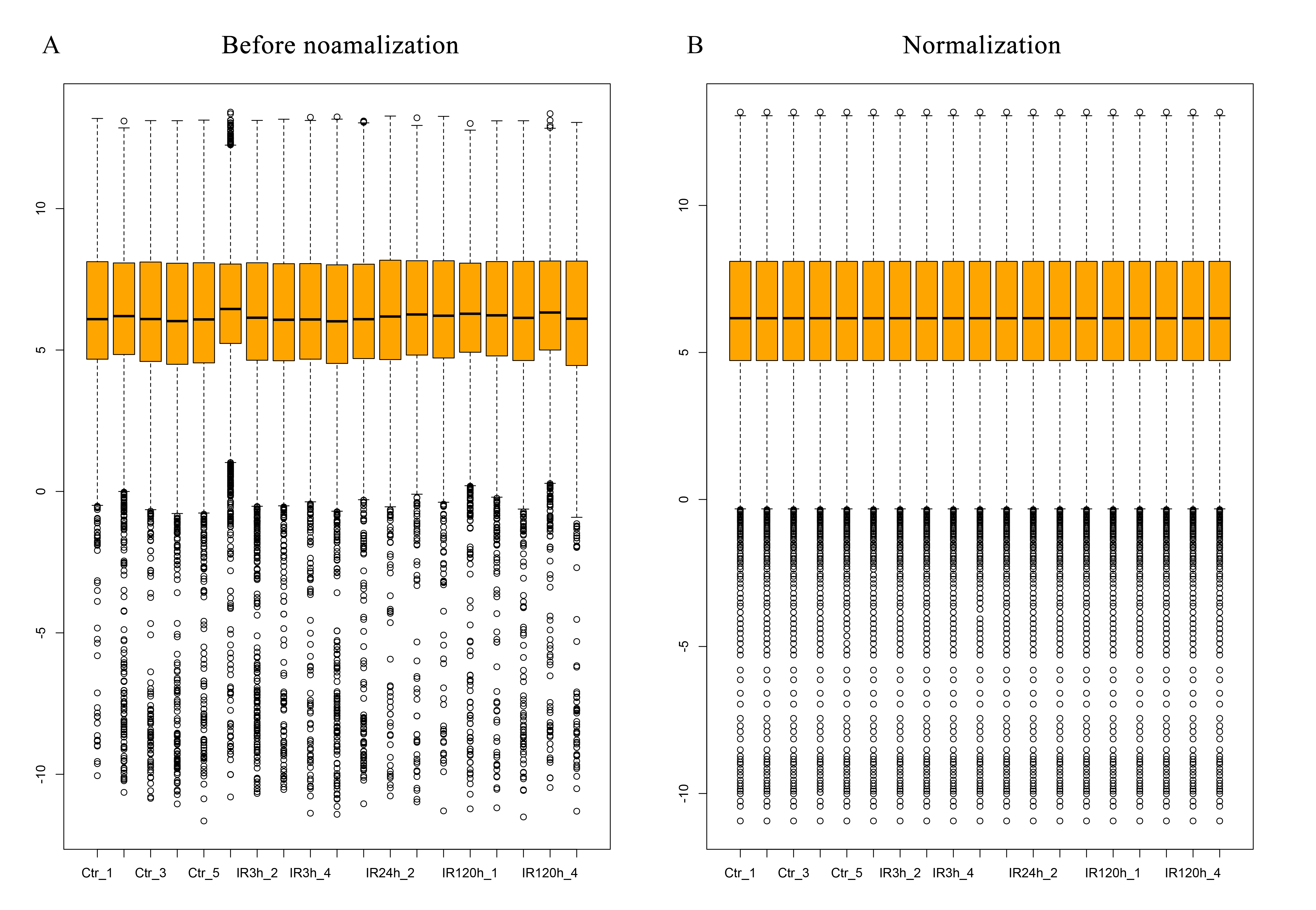

Supplement: Supplementary file 2 [file Image1.TIF]
