# Supplementary material for: miR-3587 Inhibitor Attenuates Ferroptosis Following Renal Ischemia-Reperfusion Through HO-1
Source: Front Mol Biosci. 2022 Jan 3;8:789927. doi: 10.3389/fmolb.2021.789927 (PMC8762253; doi:10.3389/fmolb.2021.789927)
Supplement: Supplementary file 3 [file DataSheet2.docx]

Table 2 Scores for the top 5 hub gene in functional cluster 1

| Sorting | Gene | Score | | | Gene | Score |
| --- | --- | --- | --- | --- | --- | --- |
|  |  | Degree | EPC | MCC |  | DMNC |
| 1 | HMOX1 | 7 | 4.351 | 840 | TIMP1 | 0.713 |
| 2 | SERPINE1 | 7 | 4.336 | 840 | LCN2 | 0.713 |
| 3 | CXCL1 | 7 | 4.329 | 840 | IL6 | 0.695 |
| 4 | MMP3 | 7 | 4.307 | 840 | HMOX1 | 0.695 |
| 5 | IL6 | 7 | 4.256 | 840 | SERPINE1 | 0.695 |

Abbreviation: EPC, Edge Percolated Component. MCC, Maximal Clique Centrality. DMNC, Density of Maximum Neighborhood Component. HMOX1, Heme Oxygenase-1. TIMP1, Tissue Inhibitor of Metalloproteinase-1. SERPINE1, Serpin Family E Member 1. LCN2, Lipocalin 2. CXCL1, C-X-C Motif Chemokine Ligand 1. MMP3, Matrix Metallopeptidase 3.
