# Supplementary material for: miR-3587 Inhibitor Attenuates Ferroptosis Following Renal Ischemia-Reperfusion Through HO-1
Source: Front Mol Biosci. 2022 Jan 3;8:789927. doi: 10.3389/fmolb.2021.789927 (PMC8762253; doi:10.3389/fmolb.2021.789927)
Supplement: Supplementary file 4 [file DataSheet1.docx]

Table 1 The details of datasets and the differential expression of HMOX1 at different time points

| Aim | Accession | Platform | Rat | Sample | Exclusion, n | Ctr, n | IR, n | IR time | adj. P-value | LogFC |
| --- | --- | --- | --- | --- | --- | --- | --- | --- | --- | --- |
| Screening | GSE58438 | GPL11534 | Wistar | Kidney | 27 | 5 | 5 | I45min/R3h | 0.00003635 | 5.31 |
|  |  |  |  |  |  |  | 4 | I45min/R24h | 0.00257 | 4.24 |
|  |  |  |  |  |  |  | 5 | I45min/R120h | 0.1052 | 1.56 |
| Verification | GSE27274 | GPL6101 | Wistar | Kidney | 0 | 6 | 6 | I20min/R6h | 0.00069931 | 2.17 |
|  |  |  |  |  |  |  | 6 | I20min/R24h | 0.00841318 | 1.52 |
|  |  |  |  |  |  |  | 6 | I20min/R120h | 0.00947 | 0.70 |
| Verification | GSE3219 | GPL2774 | SD | Kidney | 17 | 10 | 4 | I40min/R2h | 0.03042455 | 7.13 |
|  |  |  |  |  |  |  | 5 | I40min/R8h | 0.02119530 | 29.07 |
| Verification | GSE9943 | GPL2996 | SD | Kidney | 0 | 3 | 3 | I45min/R24h | 0.30506 | 2.29 |
|  |  |  | BN | Kidney | 0 | 3 | 3 | I45min/R24h | 0.99986953 | 1.44 |

Abbreviation: HMOX1, heme oxygenase-1. SD, Sprague Dawley. BN, Brown Norway. Ctr, negative control. IR, ischemia-reperfusion.
